# Evaluation of prognostic significance of lymphovascular space invasion in early stage endometrial cancer: a systematic review and meta-analysis

**DOI:** 10.3389/fonc.2023.1286221

**Published:** 2024-01-11

**Authors:** Zhao-juan Qin, Yi-si Wang, Ya-li Chen, Ai Zheng, Ling Han

**Affiliations:** ^1^ Department of Obstetrics and Gynecology, West China Second University Hospital, Sichuan University, Chengdu, China; ^2^ Key Laboratory of Birth Defects and Related Diseases of Women and Children (Ministry of Education), Sichuan University, Chengdu, China

**Keywords:** endometrial cancer, lymphovascular space invasion, recurrence, overall survival, recurrence free survival

## Abstract

**Background:**

Studies evaluating the prognostic significance of lymphovascular space invasion (LVSI) in early stage endometrial cancer (EC) are conflicting.

**Objectives:**

To evaluate whether LVSI identified in stage I EC is associated with worse survival.

**Search strategy:**

A comprehensive literature search of three databases (Embase, PubMed, and Cochrane) was performed up to April 30^th^ 2023.

**Selection criteria:**

Cohort studies that have evaluated the relationship between LVSI and prognosis in patients with stage I EC were included.

**Data collection and analysis:**

Two authors independently assessed the studies for inclusion, extracted the data of recurrence and survival, and conducted meta-analysis using random effects model. Heterogeneity was evaluated by I^2^ test.

**Main results:**

A total of 15 studies involving 6,705 patients were included in the meta-analysis. The overall pooled rate of LVSI was 14% [95% confidence interval (CI) CI 0.09-0.18] in stage I EC. LVSI was significantly associated with a higher risk of recurrence [odds ratio (OR) = 2.79, 95%CI 2.07-3.77], reduced overall survival (OS) [hazard ratio (HR)=5.19, 95%CI 3.33-8.07] and recurrence free survival (RFS) [HR = 5.26, 95%CI 3.45-8.02] in stage I EC patients. Similarly, LVSI was associated with an increased risk of recurrence [OR= 3.10, 95%CI 2.13-4.51], decreased OS [HR=5.52, 95%CI 2.16-14.09] and RFS [HR = 4.81, 95%CI 2.34-9.91] in stage IA grade 1 or 2 endometrioid carcinoma patients.

**Conclusion:**

The presence of LVSI in stage I EC and in stage IA, grade 1 or 2 endometrioid carcinoma is associated with an increased risk of recurrence, lower OS and RFS.

**Systematic Review Registration:**

https://www.crd.york.ac.uk/prospero/, identifier 42023425231.

## 1 Introduction

Endometrial cancer (EC) is the most common malignancy of the female reproductive system in developed countries, with more than 65,000 new cases reported in the United States each year (1). The prognosis of EC is influenced by various factors such as age, clinical stage, tumor differentiation, and pathological type (2). Lymphovascular space invasion (LVSI), defined as the presence of tumor cells in lymphatic or small blood vessels outside the tumor core. Up to 35% of EC patients are reported to have LVSI (3). The National Comprehensive Cancer Network (NCCN) guideliness (version 1.2023, Uterine Neoplasms) recommend quantifying LVSI to guide postoperative treatment (4). Besides, the 2023 International Federation of Gynecology and Obstetrics (FIGO) surgical staging system include LVSI in staging (5).

Nevertheless, conflicting results have been reported regarding the impact of LVSI on survival outcomes in early-stage EC. Many studies have shown that LVSI-positive is associated with lower overall survival (OS), higher recurrence, and increased risks of lymph node and distant metastasis, and is a poor prognostic factor for EC (6–8). LVSI has been reported as a risk factor, even in cases of stage I EC without lymph node metastasis (9–11). Conversely, several studies have concluded that LVSI does not significantly affect survival rates in patients with early-stage EC (12–14). For instance, Okugawa et al. (14) reported that the 5-year EC specific survival rates for tumors in LVSI-positive and LVSI-negative patients were 97.0% and 98.9%, which do not show significant difference. Furthermore, there is a lack of consensus on whether adjuvant therapy is necessary for stage I EC patients with LVSI after surgery. It is crucial to determine which EC patients with LVSI, especially those in the low-risk group (defined as stage IA, grade 1 or 2 endometrioid carcinoma), would benefit from postoperative adjuvant therapy.

Therefore, we aimed to address the knowledge gap regarding the independent prognostic significance of LVSI in early-stage EC, particularly in the low-risk group. We investigated the impact of LVSI on survival and recurrence in stage I EC. The findings of this study will contribute to improving the management and treatment decisions for stage I EC patients, aiding in the identification of those who would benefit from adjuvant therapy.

## 2 Methods

This meta-analysis was conducted in strict accordance with the Systematic Review and Meta-Analysis Preferred Reporting Project (PRISMA) statements (15), and registered in the International Register of Prospective Systems Evaluation (CRD: 42023425231).

### 2.1 Literature search

We performed a comprehensive literature search of three databases: Embase, PubMed, and Cochrane, from database inception to April 30th 2023. Cohort studies that have evaluated the relationship between LVSI and prognosis in patients with stage I EC were included. The predefined search string was the following: (endometrial cancer OR endometrial carcinoma OR uterine cancer OR uterine carcinoma of endometrium) AND (early stage OR stage IA OR stage I OR FIGO I OR FIGO IA) AND (lymphovascular space invasion OR LVSI). Other literature resources, such as the reference lists of eligible studies, were reviewed to identify additional studies. We applied language restriction that only publications in English were included. There were no restrictions on the publication time, publication status or article type.

### 2.2 Study selection

Two authors (ZJ Qin and YS Wang) independently screened the titles and abstracts based on the inclusion criteria to determine relevant studies. After initial screening, the full text of all potentially eligible articles were independently reviewed by two authors for further assessment. Any discrepancies were resolved through discussion with the corresponding author.

The inclusion criteria for our research were as follows: (a) studies that are either prospective or retrospective cohort studies; (b) inclusion of EC patients who have undergone surgical pathological staging, FIGO stage I or IA, and any pathological type; (c) analysis of the prognosis of patients with and without LVSI; and (d) availability of the full text. The exclusion criteria were as follows: (a) inability to extract valid outcome indicators from the literature; (b) non-English literature; (c) low scores in quality assessment; and (d) sample size less than 50.

### 2.3 Data extraction

Two authors (ZJ Qin and YL Chen) independently extracted the following data from each study: first author name, publication year, country, inclusion of EC staging and pathological type, time of initial treatment, median follow-up time, LVSI incidence, administration of adjuvant therapy, and outcome data related to the potential relationship between LVSI and prognosis, including recurrence, OS, and recurrence-free survival (RFS). Any discrepancies in data extraction were resolved through discussion with the corresponding author.

### 2.4 Quality assessment

The methodological quality of the studies included in the research was independently assessed by two authors (ZJ Qin and A Zheng) according to the Newcastle-Ottawa Quality Assessment Scale (NOS) (16). For the ‘comparability of cohort based on design or analysis’ criterion, studies that controlled for histological type of endometrioid adenocarcinoma received 1 star. For the ‘adequacy of follow-up time in the cohort’ criterion, a median follow-up time of more than 40 months was rated 1 star. Studies scoring 6 stars or more were considered high-quality and were included in our meta-analysis.

### 2.5 Statistical analysis

Statistical analysis was conducted using the metan, metabias, and metaprop software packages in STATA 15.0 (Statacorp, College Station, TX, USA). We calculated the odds ratio (OR) of recurrence, the hazard ratio (HR) of RFS and OS, as well as their 95% confidence intervals (Cls). In studies that only reported OS and RFS as Kaplan-Meier curves and where HR and 95% intervals could not be obtained from the original text, the necessary data were extracted using engage Digitizer 4.1 (http://sourceforge.net/projects/digitizer/), and HR was calculated using the tool recommended by Tierney et al (17).

Heterogeneity was quantified using the I^2^ statistic: I^2^ <30% was considered low heterogeneity, 30% <I^2^ <50% was considered moderate heterogeneity, and I^2^ >50% was considered high heterogeneity (18). Given the clinical heterogeneity of the studies included, we used a random-effects model for all meta-analyses. We created Galbraith radial plots to explore potential causes of heterogeneity. We also conducted subgroup analyses based on FIGO stage, histological type, and grade of differentiation. The Begg’s test was used to assess the risk of publication bias, with a *p*-value of less than 0.1 considered as evidence of significant publication bias.

## 3 Results

### 3.1 Characteristics of the included studies

**Figure 1:**
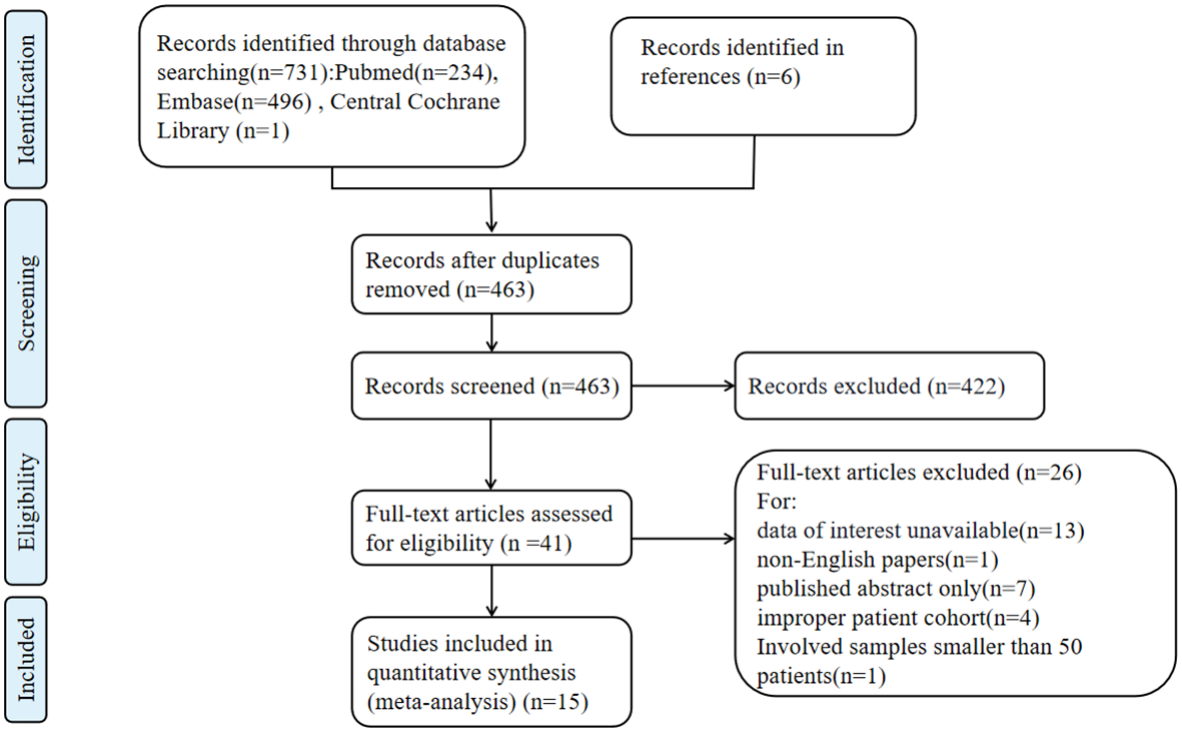
Flow diagram of literature searching and study selection.

A total of 731 records were identified through the literature search. After removing duplicates, 463 articles underwent title and abstract screening, and 41 articles were selected for full-text screening. Ultimately, 15 studies (3, 6–14, 19–23) involving 6705 patients were included in the data analysis. The characteristics of the 15 included studies are shown in **Table 1**. The study selection process is illustrated in **Figure 1**. These studies were published between 2007 and 2022. All the included studies achieved a Newcastle-Ottawa Scale (NOS) score of 6 or higher, with the highest score being 8.

**Table 1:** Characteristics of studies included in the meta-analysis.

Study	Country	Study type	Inclusion criteria	Pathological type	N	LVSI+	LVSI-	Median follow-up	Quality assessment*
Okugawa(2021)	Japan	R	IA	mixed	297	35	262	60m	7
Iida(2022) (12)	Japan	R	IA	mixed	116	15	101	71.9m	7
Tortorella(2021) (3)	Italia	R	IA G1/G2	Endometrioid carcinoma	524	57	467	38m	7
Reis(2015) (20)	America	R	IA G1/G2	Endometrioid carcinoma	200	40	200	46.6m	8
Nwachukwu(2021) (11)	America	R	IA G1	Endometrioid carcinoma	222	14	204	20m	7
Güngördük(2018) (21)	Turkey	R	IA G1/G2	Endometrioid carcinoma	280	22	258	54-69m	8
Ayhan(2019) (19)	Turkey	R	IA G1/G2	Endometrioid carcinoma	912	53	859	42m	8
Ureyen(2019)	Turkey	R	IA	Endometrioid carcinoma	720	60	660	48m	8
Pifer(2022)	Pittsburgh	R	I	Endometrioid carcinoma	335	124	211	25.8m	7
Veade(2019) (7)	America	R	I	Endometrioid carcinoma	275	48	227	54m	8
Cusano(2018) (9)	Canada	R	I	Endometrioid carcinoma	400	54	346	66m	8
Bosse(2015) (22)	Netherlands	R	I	Endometrioid carcinoma	926	70	856	89-160m	8
Aristizabal (2014) (23)	France	R	I	mixed	384	112	272	38.7m	6
Gemer(2007) (10)	Israel	R	I	mixed	699	40	659	39m	6
Yarandi(2023) (6)	Iran	R	I G1/G2	Endometrioid carcinoma	415	100	315	NM	6
Total					6705	844	5897		

R, retrospective; G1/G2, grade1/2; NM, not mentioned.

*Quality assessment was measured using the Newcastle-Ottawa Quality Assesment Scale (NOS).

### 3.2 Rate of LVSI

**Figure 2:**
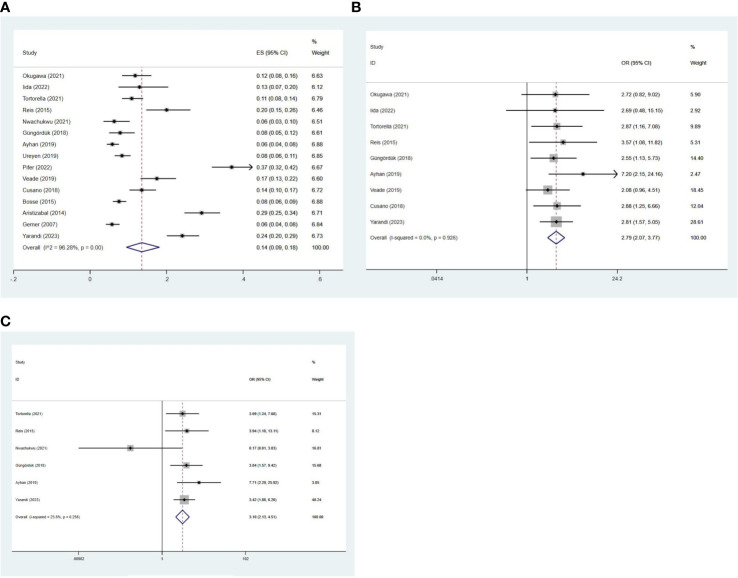
Forest plots of the **(A)** incidence of LVSI; potential relationships of LVSI with recurrence in **(B)** stage I and **(C)** stage IA G1/2 EC.

A total of 15 studies involving 6,705 patients investigated the incidence of LVSI in early-stage EC. The pooled incidence of LVSI was 14% (95% CI 0.09-0.18; I^2^ = 96.28%, *p* < 0.001) in stage I EC (**Figure 2A**). Given the high heterogeneity observed among the LVSI meta-analyses of all studies, subgroup analyses were conducted based on country, sample size, publication year, and pathological subtype. However, these subgroup analyses did not clearly identify the source of heterogeneity, and further analysis of individual study characteristics failed to explain the observed heterogeneity.

### 3.3 Relationship between LVSI and recurrence

Nine studies (3, 6–9, 11, 12, 14, 21) involving 3,337 patients evaluated the relationship between LVSI and recurrence. The recurrence rate for patients with LVSI was 18.10% (72/398), compared to 6.16% (181/2939) for patients without LVSI in stage I EC. The meta-analysis showed that patients with LVSI had a significantly higher risk of recurrence (OR = 2.79, 95%CI 2.07-3.77; I^2^ = 0%, *p* = 0.926) in stage I EC (**Figure 2B**).

Further analysis revealed that the recurrence rate for patients with LVSI was 14.02% (30/214), compared to 5.03% (108/2148) for patients without LVSI in stage IA grade 1 or 2 endometrioid carcinoma. Similarly, patients with LVSI also had a significantly higher recurrence rate (OR = 3.10, 95%CI 2.13-4.51; I^2^ = 23.8%, *p* = 0.256) in stage IA grade 1 or 2 endometrioid carcinoma (**Figure 2C**).

### 3.4 Relationship between LVSI and survival

**Figure 3:**
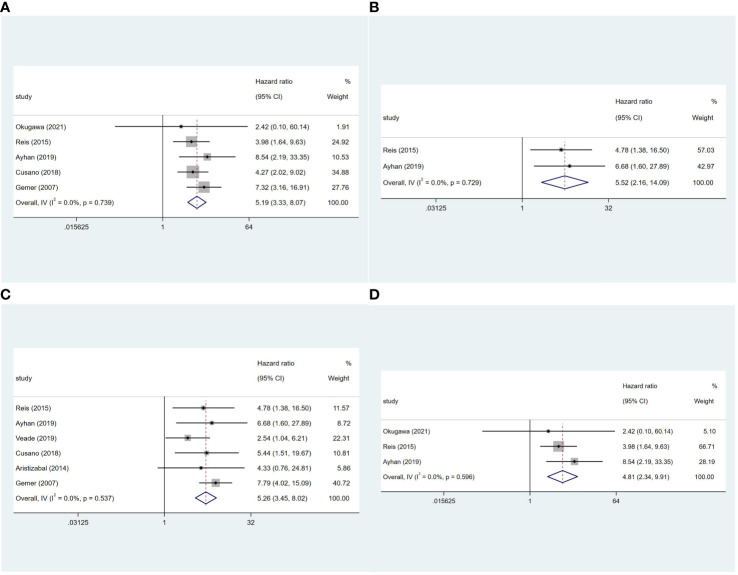
Forest plots of the potential relationships of LVSI with **(A)** overall survival in stage I and **(B)** stage IA G1/2 EC; **(C)** recurrence-free survival in stage I and **(D)** stage IA G1/2 EC.

Five studies (8–10, 14, 20) reported on the OS of 2,880 patients. LVSI-positive patients in stage I EC had a 5.19-fold higher risk of death than LVSI-negative patients (95%CI 3.33-8.07; I^2^ = 0, *p* = 0.739; **Figure 3A**). In stage IA grade 1 or 2 endometrioid carcinoma patients, LVSI-positive had a 5.52-fold higher risk of death than LVSI-negative patients (95%CI 2.16-14.09; I^2^ = 0, *p* = 0.729; **Figure 3B**). Additionally, six studies (8–10, 20, 23) reported on the RFS of 2,595 patients. LVSI significantly influenced RFS in stage I EC (HR = 5.26, 95%CI 3.45-8.02; I^2^ = 0, *p* = 0.537; **Figure 3C**) and stage IA grade 1 or 2 endometrioid carcinoma patients (HR = 4.81, 95%CI 2.34-9.91; I^2^ = 0, *p* = 0.596; **Figure 3D**).

### 3.5 Publication bias

**Figure 4:**
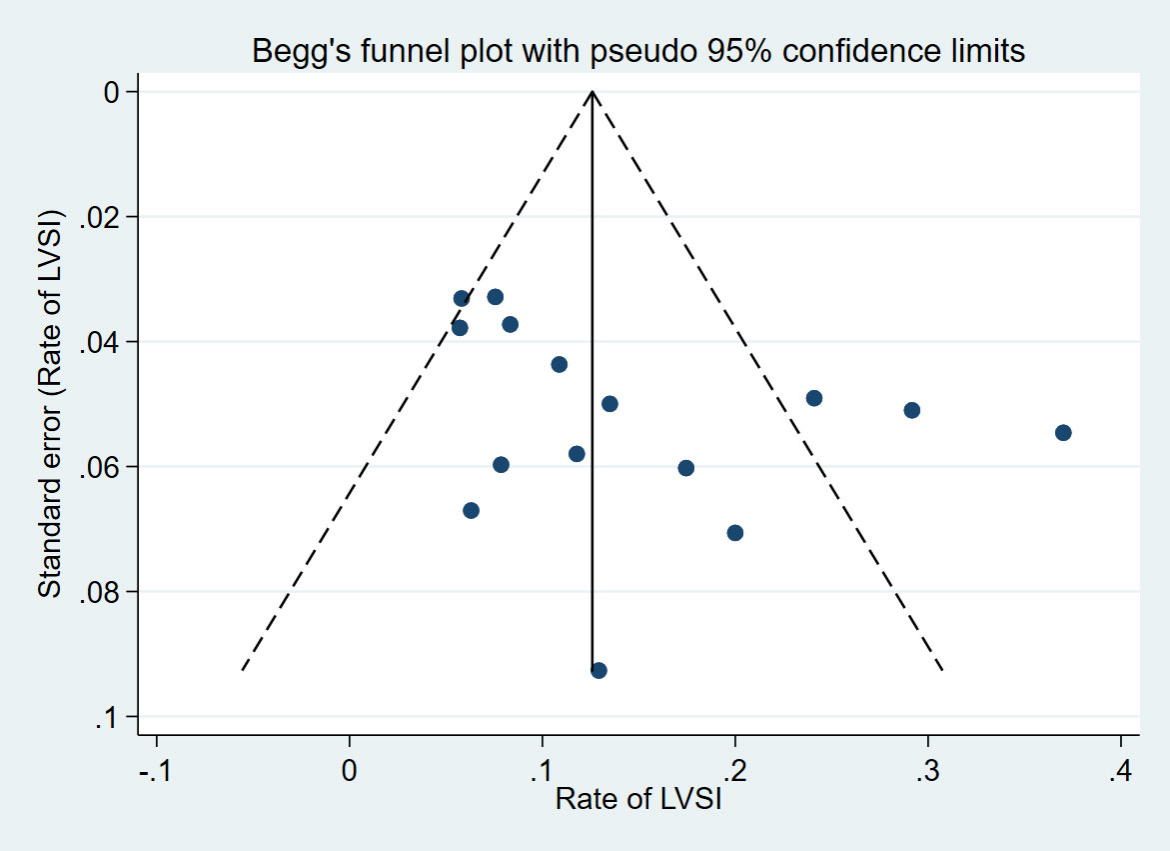
Funnel plot with pseudo 95% confidence limits of 15 included studies reporting the rate of LVSI.

Publication bias was assessed based on the LVSI rate and was reported in most of the included studies. Ultimately, 15 studies were included, and the Begg’s test (*p*=0.067) showed a potential source of publication bias. According to our funnel plot, studies with a lower LVSI rate are more likely to be published (**Figure 4**).

## 4 Discussion

Our analysis revealed that approximately 14% (95% CI 0.09-0.18) of stage I EC patients had LVSI, consistent with the reported range of LVSI frequency in the literature ranging from 6% (8) to 37% (13). This variability may be attributed to the inclusion criteria that did not limit the pathological type of EC, encompassing both endometrioid adenocarcinoma and other types of EC. Additionally, the clinical stages included both stage IA and stage I, introducing clinical heterogeneity in LVSI positive rates.

Importantly, our findings demonstrate that LVSI is significantly associated with a higher risk of recurrence, not only in stage I EC (OR= 2.79, 95%CI 2.07-3.77) but also in stage IA grade 1 or 2 endometrioid carcinoma patients (OR= 3.10, 95%CI 2.13-4.51). These findings are in line with previous investigations (3, 9, 10, 24), suggesting that the presence of LVSI increases the risk of tumor recurrence. Additionally, LVSI was found to significantly influence RFS in both stage I EC (HR = 5.26, 95%CI 3.45-8.02) and stage IA grade 1 or 2 endometrioid carcinoma patients (HR = 4.81, 95%CI 2.34-9.91). The presence of LVSI in early endometrial cancer patients increases the risk of tumor recurrence, Bosse et al. (22) and Güngördük et al. (21) illustrated the presence of LVSI was an independent risk factor for recurrence. Besides, LVSI is independently associated with lymph node metastasis in women with early-stage endometrial cancer (25).

Furthermore, our meta-analysis demonstrated that patients with LVSI have worse OS in both stage I EC (HR=5.19, 95%CI 3.33-8.07) and stage IA grade 1 or 2 endometrioid carcinoma patients (HR=5.52, 95%CI 2.16-14.09) compared to patients without LVSI. Our research findings are in concordance with previous studies reporting a significant association between LVSI and decreased OS (3, 6, 7). However, it is important to note that there have been conflicting reports regarding the impact of LVSI on prognosis. For instance, Iida et al. (12) found no difference in prognosis between patients in stage IA and type 1 EC with and without LVSI, Okugawa et al. (14) reported that LVSI did not significantly affect cancer-specific survival or serve as a prognostic factor in stage IA endometrial cancer. The reason for this difference may be the specific study design that included the stage of early EC, pathological type, and the proportion of lymph nodes evaluated. The discrepancies in these findings highlight the need for further research to explore the prognostic value of LVSI in early EC.

It is crucial to determine which EC patients, especially those in the low-risk group, would benefit from postoperative adjuvant therapy. There is a lack of consensus on whether adjuvant therapy is necessary for stage I EC patients with LVSI after surgery. Notably, studies by Beavis et al. (26) and Son et al. (27) reported improved progression-free survival with adjuvant therapy compared to observation alone in stage I endometrioid EC patients with LVSI. However, none of the four randomized studies (28–31) showed that adjuvant radiotherapy improved survival in stage I EC patients. In fact, Johnson et al. (32) reported that external beam radiation therapy (EBRT) after surgery reduced the risk of local recurrence, but did not reduce the risk of distant metastasis or OS in stage I EC. Thus, the role of adjuvant therapy in LVSI subgroups of early-stage EC remains uncertain and requires further investigation. Our study shows that LVSI increases the risk of early EC recurrence and death. Postoperative observation and follow-up can be selected for endometrioid adenocarcinoma with LVSI in the low-risk group of stage IA, but EBRT supplementation may be considered when combined with other high-risk factors such as age over 60 years old, lesion diameter greater than 2cm or molecular classification indicating poor prognosis. Other stage I EC patients with LVSI are advised to consider EBRT.

This study also has certain limitations. First, clinical heterogeneity exists among the included studies due to differences in pathology types, surgical procedures, and adjuvant treatment methods. Second, the retrospective nature of the included studies introduces a risk of selection bias. Peters et al (33). have studied the correlation between the extent of LVSI and prognosis in patients with EC using samples from PORTEC trials. Their quantitative analysis revealed a significant correlation between the extent of LVSI and the risk of pelvic lymph node recurrence in EC patients. They propose defining clinically relevant LVSI in EC as the involvement of more than 4 LVSI-positive vessels in at least one H&E slide. However, due to insufficient LVSI grading data in the included literature, we were unable to perform subgroup analysis based on this parameter. Additionally, there may also be inconsistent diagnostic criteria for LVSI positivity, leading to potential confounding bias in relevant studies. We only included English-language literature and publications and may have a publication bias risk.

In conclusion, our analyses demonstrates that the presence of LVSI in stage I EC and in stage IA, grade 1 or 2 endometrioid carcinoma is associated with an increased risk of recurrence, reduced RFS and OS. LVSI status should be considered as an important prognostic factor in early stage EC, and its presence should prompt closer postoperative clinical follow-up. The role of adjuvant therapy in LVSI subgroups of early-stage EC remains controversial and requires further investigation. Future research should focus on evaluating the survival benefits and potential risks of adjuvant radiotherapy in patients with LVSI to determine its optimal use in clinical practice.

## Data availability statement

The original contributions presented in the study are included in the article/supplementary files, further inquiries can be directed to the corresponding author/s.

## Author contributions

Z-JQ: Conceptualization, Data curation, Formal Analysis, Investigation, Methodology, Project administration, Software, Supervision, Validation, Writing – original draft. Y-SW: Data curation, Investigation, Methodology, Software, Writing – original draft. Y-LC: Formal Analysis, Investigation, Methodology, Software, Writing – original draft. AZ: Formal Analysis, Supervision, Visualization, Writing – review & editing. LH: Conceptualization, Formal Analysis, Supervision, Validation, Writing – review & editing.
